# Antibacterial Additives in Epoxy Resin-Based Root Canal Sealers: A Focused Review

**DOI:** 10.3390/dj7030072

**Published:** 2019-07-01

**Authors:** Alexander Brezhnev, Prasanna Neelakantan, Ray Tanaka, Sergey Brezhnev, George Fokas, Jukka P. Matinlinna

**Affiliations:** 1 Applied Oral Sciences—Dental Materials Science, Faculty of Dentistry, The University of Hong Kong, Hong Kong, China; 2 Discipline of Endodontology, Faculty of Dentistry, The University of Hong Kong, Hong Kong, China; 3 Applied Oral Sciences—Oral and Maxillofacial Radiology, Faculty of Dentistry, The University of Hong Kong, Hong Kong, China; 4 Department of Prosthodontics, Faculty of Dentistry, The University of Hong Kong, Hong Kong, China

**Keywords:** antimicrobial, biofilm, endodontics, epoxy resin, nanoparticle, quaternary ammonium, obturation, root canal, sealer

## Abstract

Dental materials used in root canal treatment have undergone substantial improvements over the past decade. However, one area that still remains to be addressed is the ability of root canal fillings to effectively entomb, kill bacteria, and prevent the formation of a biofilm, all of which will prevent reinfection of the root canal system. Thus far, no published review has analysed the literature on antimicrobial additives to root canal sealers and their influence on physicochemical properties. The aim of this paper was to systematically review the current literature on antimicrobial additives in root canal sealers, their anti-fouling effects, and influence on physicochemical properties. A systematic search was performed in two databases (PubMed and Scopus) to identify studies that investigated the effect of antimicrobial additives in epoxy resin-based root canal sealers. The nature of additives, their antimicrobial effects, methods of antimicrobial testing are critically discussed. The effects on sealer properties have also been reviewed. A total of 31 research papers were reviewed in this work. A variety of antimicrobial agents have been evaluated as additives to epoxy resin-based sealers, including quaternary ammonium compounds, chlorhexidine, calcium hydroxide, iodoform, natural extracts, antibiotics, antifungal drugs, and antimicrobial agent-functionalised nanoparticles. Antimicrobial additives generally improved the antimicrobial effect of epoxy resin-based sealers mainly without deteriorating the physicochemical properties, which mostly remained in accordance with ISO and ANSI/ADA specifications.

## 1. Introduction

Optimal disinfection and a fluid-tight seal of the root canal system are considered important cornerstones for the success of endodontic treatment. While the exact reason for the failure of root canal treatment could be multifactorial, remnant microbes within the complex anatomy appear to be the predominant causative factor [1,2]. Bacterial persistence after chemo-mechanical disinfection influences the outcome of treatment [3]. The microbial aetiology of endodontic infections and reinfections is complex. Although the root canal microbiome is polymicrobial, *Enterococcus faecalis*, a Gram-positive coccus, appears to be the most commonly isolated bacterium from persistent lesions [1]. This may be attributed to the ability of this organism to survive extremely harsh environmental conditions, adhere to the root canal dentine, and form biofilms, which can be 1000-fold more resistant to disinfection strategies, compared to the planktonic state of these bacteria [2,4]. This organism is specifically interesting due to its important role in nosocomial infections and resistance to multiple antibiotics.

Root canal obturation is considered by some authors as a “second phase” of endodontic treatment the sole aim of which is to seal the canal space and prevent reinfection. According to this approach, the root canal filling material does not need to possess antibacterial properties as the disinfection of the root canal system is accomplished in the “microbial control phase” [5]. There are numerous approaches and disinfection strategies to increase the effectiveness of this treatment phase [6,7].

Even with advances in materials and technology, it is well realized that achieving a sterile root canal system is extremely challenging, if not impossible [2]. Moreover, even when root canal treatment is consistent with high standards, failure may occur due to resistant intraradicular biofilm [8,9,10,11].

This said, microbes that remain within the eccentric and complex anatomy of the root canal system need to be entombed and possibly killed or rendered avirulent by root canal sealers, to prevent reinfection. All root canal sealers appear to have some degree of intrinsic antimicrobial activity owing to their composition [12,13,14,15]. However, this effect is time-dependent, and it remains unknown whether this can prevent reinfection of the root canal system in the long term. Nevertheless, modifying these materials with antimicrobial additives that prevent biofilm formation is worthwhile from the context of preventing reinfection. While a variety of sealers are available currently, epoxy resin (epoxy resins a.k.a. polyepoxides are a class of reactive prepolymers with epoxide functionality) sealers are among the most widely used due to their adequate sealing ability, physicochemical, biological properties [16,17,18,19], and the possibility of chemical bonding to dentinal collagen [20].

Over the past few years, attempts have been made to modify root canal sealers with antimicrobial nanoparticles [21,22], antibiotics [23,24,25,26,27,28,29], antiseptics [30,31,32,33,34] with the aim of having minimal or no impact on the physicochemical and biological properties apart from the antimicrobial activity. A wide-spread interest was evident in modifying sealers with antibacterial additives, but the main problem was a lack of standardized approach in antimicrobial testing. Thus far, the modifications of root canal sealers to demonstrate antimicrobial activity and the resultant effect on their physicochemical properties have not been reviewed or published. The aim of this focused review was to identify the effects of antimicrobial additives on the properties of epoxy resin-based sealers. The present study examined the influence of intervention on existing: (a) antimicrobial effects and (b) physicochemical properties of epoxy resin-based sealers. The existing body of literature was systematically searched and critically discussed. This review offers significant insights into the scope of modification of root canal fillings. It will help establish a synergy between material scientists and clinicians to develop novel anti-biofouling materials that will prevent reinfection of the root canal system.

## 2. Review

### 2.1. Search Strategy

The study was constructed based on the PICO principle: Population (root canal reinfection), Intervention (modification of epoxy resin root canal sealers with antimicrobial additives), Comparison (pure epoxy resin sealer), and Outcome (antimicrobial effectiveness). The research question based on the PICO approach was: Does modification of an epoxy resin root canal sealer with antimicrobial additives significantly improve its antimicrobial properties compared to a pure epoxy resin sealer?

Although this work is a focused review, we performed the search strategy in a systematic manner. Terms and keywords relating to endodontics, root canal filling, and antimicrobials were used to search for potential articles, with no restriction of publications. The search was conducted in March 2019 in two electronic databases, PubMed and Scopus, and the search strategy was modified based on the database used. A representative search strategy in PubMed and Scopus is shown in Table 1 and Table 2, respectively.

In addition, hand searching was performed in the main journals related to endodontics and dental materials science (Australian Endodontic Journal, Clinical Oral Investigations, Dental Materials, Dental Materials Journal, International Endodontic Journal, Journal of Dental Research, Journal of Dentistry and Journal of Endodontics) to identify articles that were possibly not found in the electronic databases. The electronic portals of each of these journals were searched to identify any additional articles (early view/in press or accepted). The search strategy followed the PRISMA (Preferred Reporting Items for Systematic reviews and Meta-Analyses) guidelines [35].

### 2.2. Eligibility Criteria

Titles and abstracts of the identified articles were appraised to remove articles that were out of scope. Reviews were also excluded. Full texts of the remaining articles were obtained for further analysis. References of the selected articles were searched for any potential articles to be included. Studies that investigated the antimicrobial properties of epoxy resin root canal sealers incorporated with antimicrobial agents were included for further review. Additionally, studies which investigated the influence of antimicrobial additives on physicochemical properties of epoxy resin sealers were also included. Only publications in the English language were included in this review. The above analyses were done by two independent reviewers (A.B., P.N.) and in the case of disagreement, the consensus was reached after discussion with the third reviewers (J.P.M.). The primary outcome measure being reviewed was antimicrobial efficacy or the antibiofilm effect of modified sealers, while the secondary outcome measure was the physicochemical properties of the modified sealers.

### 2.3. Results

**Figure 1:**
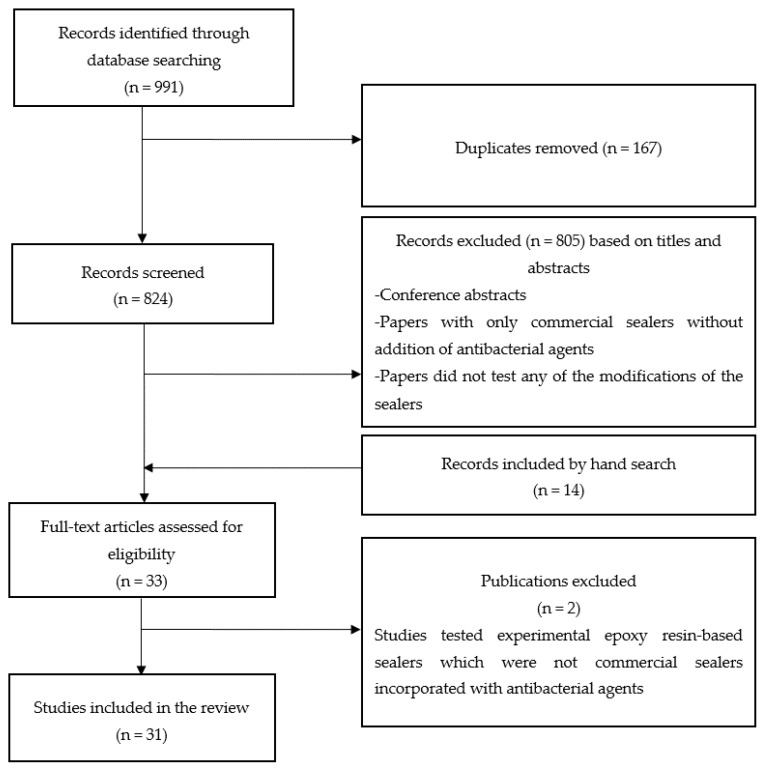
PRISMA (Preferred Reporting Items for Systematic reviews and Meta-Analyses) flowchart showing the search process.

**Figure 2:**
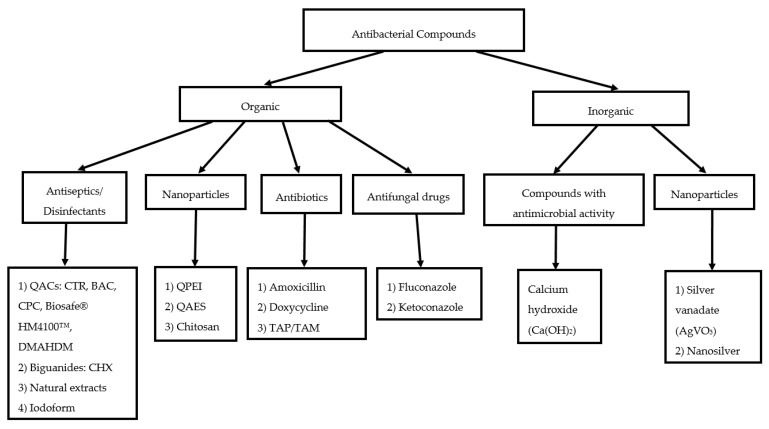
Classification of Antibacterial Compounds Discussed in the Review. Key: QACs = quaternary ammonium compounds; CTR = cetrimide; BAC = benzalkonium chloride; CPC = cetylpyridinium chloride; Biosafe^®^ HM4100™ = 3-(trihydroxysilyl)-propyldimethyloctadecyl ammonium chloride; DMAHDM = dimethylaminododecyl methacrylate; CHX = chlorhexidine digluconate; QPEI = quaternary ammonium polyethylenimine; QAES = quaternary ammonium epoxy silicate; TAP/TAM = triple antibiotic paste/mixture consisting of (minocycline or doxycycline) + metronidazole + ciprofloxacin.

The search yielded a total of 991 papers. After the removal of duplicates (n = 167), 824 records were screened for eligibility. A total of 31 papers were included for full-text review. The search strategy is summarized in Figure 1. Classification of the antibacterial additives discussed in the review is shown in Figure 2. Of the 31 papers included, 11 [31,32,36,37,38,39,40,41,42,43,44] evaluated the effect of antiseptic additives, including natural agents (plant extracts) [42,43], two publications tested calcium hydroxide as an additive [45,46], 5 [23,24,25,28,47], and one [48] evaluated the effects of antibiotic and antifungal additives, respectively. Nanoparticles and/or nanoparticles functionalised with antimicrobial agents were studied in 12 papers [21,49,50,51,52,53,54,55,56,57,58,59]. The epoxy resin-based sealers used in the studies were AH Plus™ (Dentsply DeTrey, Konstanz, Germany) [23,24,25,31,32,38,39,40,41,42,43,45,46,47,48,49,50,51,52,53,54,56,57,58,59], AH 26™ (Dentsply DeTrey, Konstanz, Germany) [24,28,57], Sealer 26™ (Dentsply, Indústria e Comércio Ltda., Petrópolis, RJ, Brazil [48,50,51,52], Dentsply, Maillefer Instruments SA, Ballaigues, Switzerland [44]), ThermaSeal Plus™ (Dentsply Tulsa Dental, Tulsa, OK, USA) [21], MM-Seal ™ (MicroMega, Besançon, France) [36], BJM Root Canal Sealer™ (B.J.M. Laboratories, Or-Yehuda, Israel) [36,37], and RCS™ (B.J.M. Laboratories, Or-Yehuda, Israel) [55,57]. The findings of the included studies have been summarized in Table 3.

## 3. Review of Antimicrobial Effects

### 3.1. Antiseptics/Disinfectants and Compounds with Antimicrobial Activity

#### 3.1.1. Quaternary Ammonium Compounds (QACs)

Quaternary ammonium compounds (QACs) are effective antimicrobial agents used also in dental materials [60,61,62]. The effect of addition of QACs such as benzalkonium chloride (BAC; alkyldimethylbenzylammonium chloride), cetylpyridinium chloride (CPC), and cetrimide (CTR; a blend of quaternary ammonium salts and cetrimonium bromide) were tested using standard microbiological methods such as the agar diffusion (Kirby-Bauer) test [39], direct contact test [31], as well as more robust antibiofilm experiments [31,40]. An important consideration in this regard is that the size of the inhibition zones does not provide a direct correlation of the antimicrobial effect due to the influence of the diffusion of certain components from the sealer through the agar medium [39]. It is, therefore, recommended to avoid using this test for antibacterial evaluation [63]. The authors [39] attempted to minimize the effect of differences in the material by using a 24 h pre-diffusion time before the sealers were completely set (polymerized). From these tests, it appears that the AH Plus™ modified with the 2% BAC or CPC demonstrated significantly larger zones of inhibition than the unmodified version of the same material, against *Streptococcus mutans* and *Lactobacillus casei*. Furthermore, this activity decreased with time. While unmodified AH Plus™ showed no inhibition zones against *S. mutans* after 7 days and 21 days, the modified sealer’s inhibition zones were larger. All versions of the sealer were most effective against *Actinomyces viscosus*, although the effect decreased significantly after 7 days and 21 days.

Using a combination of Direct Contact Test (DCT) and antibiofilm experiments, Arias-Moliz et al. [40] evaluated the effectiveness of 1 w/w %, 2 w/w %, and 3 w/w % benzalkonium chloride added to AH Plus™. They determined that 3% showed a total killing of *Enterococcus faecalis* in the DCT, but there was no significant difference between 2% and 3% BAC in the assessment of growth kinetics (Colony forming units, CFU). However, both 2% and 3% BAC reduced the CFU/mL significantly higher than 1% BAC and unmodified AH Plus™. Biofilm analysis was performed using confocal laser scanning microscopy (CLSM), in the Calgary biofilm device (MBEC-high throughput [HTP]; Innovotech, Edmonton, Alberta, Canada). CLSM demonstrated that addition of 2% and 3% BC significantly reduced the total biomass formed on the sealer surface, with the latter showing significantly less biovolume than the former. The addition of 3% BAC decreased the biovolume by more than 30 times compared with pure AH Plus™. Interestingly, only 57.6% of bacteria on the surface of pure AH Plus™ sealer were alive. Although 3% BAC prevented microbial adhesion to its surface via electrostatic repulsion, 70.9% of bacteria that adhered to the surface were alive. The advantage of CLSM is that it allows a 3-dimensional representation of the biofilm along with the ability to image live and dead microbes using appropriate stains. These results demonstrate the need for further research on the ability of BAC to disrupt or prevent biofilms in a more clinically relevant root canal model.

Another quaternary ammonium compound tested as an additive to the AH Plus™ sealer was cetrimide (CTR) in combination with chlorhexidine digluconate (CHX). CTR 0.1%, 0.2%, 0.3%, 0.4%, or 0.5% alone in AH Plus™ or combinations with 1% or 2% CHX were tested by the modified direct contact test (DCT) or against biofilm formation. *E. faecalis* biofilm formation was studied using the Calgary biofilm device (MBEC-high throughput [HTP]; Innovotech, Edmonton, AB, Canada) [31]. This study showed that the antibacterial and antibiofilm effects of the modified AH Plus™ were dependent on the concentration of the additives. AH Plus™ mixed with 1% or 2% CHX resulted in higher antibacterial effect than AH Plus™ alone. The inhibition of biofilm formation depended on the CHX concentration in AH Plus™, though no concentration could eradicate biofilm formation. CTR at 0.2% concentration was also able to inhibit *E. faecalis* biofilm formation. Interestingly, the DCT approach showed that eradication of *E. faecalis* was possible with 0.3% CTR and higher combined with 2% CHX. The authors also reported that CTR, in concentration as little as 0.1% combined with any of CHX concentrations was enough to inhibit biofilm formation [31]. These data demonstrate that the addition of QACs to AH Plus™ does have an impact on antimicrobial activity. However, the method of analysis needs to be more robust to generate clinically relevant data. Furthermore, the time-dependent effects of the QACs remain to be investigated.

Becker et al. [37] tested the addition of another quaternary ammonium compound Biosafe^®^ HM4100™ (3-(Trihydroxysilyl) propyldimethyloctadecyl ammonium chloride) at 0%, 0.4%, 0.8%, 1.6%, 3.3% w/v into BJM Root Canal Sealer™. It is a silicon-based polymer that possesses a positive charge and antibacterial properties due to an ammonium cation in its molecular structure. In their protocol, they prepared a 48 h biofilm of *E. faecalis*, applied crystal violet stain and employed a spectrophotometer to read optical density of further diluted stain eluate. Authors stated significant reduction in new biofilm formation of 25% and 72% in 1.6% and 3.3% w/v groups, respectively. By performing another test against formed biofilm of *E. faecalis* the authors used fluorescence microscopy images and concluded on the biofilm viability that there was a significant reduction of 20% and 36% in 1.6% and 3.3% w/v groups.

Seung et al. [41] investigated not only the addition of dimethylaminododecyl methacrylate (DMAHDM) at 2.5%, 5%, 10% w/w, but also a combination with nanosilver (NAg) at 0.15% w/w into AH Plus™. First, the authors tested each of the compounds separately mixed with AH Plus™ in order to decide on optimal combination when used together. Based on the preliminary tests, a mixture of 2.5% DMAHDM and 0.15% was chosen due to good results in physicochemical tests. AH Plus™ with only 0.15% NAg caused a significantly higher bacterial reduction on day 1 in comparison with the pure AH Plus™, but there was no difference between them at days 7 and 14. The mean CFU/mL values for AH Plus™ with 2.5% DMAHDM and AH Plus™ with 2.5% DMAHDM and 0.15 NAg were significantly lower compared with the control at each time point (day 1, day 7, day 14). It is worth noting that the incorporation of these additives promoted significantly higher antibacterial effect even after 14 days compared with the pure AH Plus™.

#### 3.1.2. Natural Agents

Hinokitiol, 4-isopropyltropolone (2-Hydroxy-6-propan-2-ylcyclohepta-2,4,6-trien-1-one), a natural monoterpenoid found in the *Cupressaceae* tree family, is used as a topical drug. This monoterpenoid has been evaluated as a possible additive in AH Plus™. Hinokitiol has a potent antimicrobial effect and has also been used in toothpastes and oral gels to manage the oral lichen planus and reduce halitosis [42]. Unmodified AH Plus™ showed clear inhibition zones against methicillin-resistant *Staphylococcus aureus* (MRSA) by agar diffusion test (ADT), the limitations of which were discussed earlier. The inhibition zone diameters increased after the addition of 0.2% hinokitiol. A direct contact test (DCT) revealed that the growth of MRSA was completely inhibited by either AH Plus™ alone or with the addition of hinokitiol 0.2% [42]. Hinokitiol did not improve the antibacterial properties of AH Plus™ against MRSA in the DCT, probably due to a strong inherent effect of AH Plus™ against this pathogen. Further research is needed to understand the effects of this agent on endodontically relevant pathogens in a biofilm model. It would be worth testing this compound against *E. faecalis* and other pathogens to better understand its activity.

Saha et al. [43] mixed three herbal extracts—*Glycyrrhiza glabra* (Licorice), *Tinospora cordifolia* (Guduchi), *Mimusops elengi* (Bakul)—with AH Plus™ and compared zones of inhibition in ADT against five aerobes and facultative anaerobes (*Staphilococcus aureus, Streptococcus ß haemolyticus*, *Enterococcus faecalis*, *Escherichia coli*, *Pseudomonas aeruginoca*) and two obligate anaerobes (*Peptostreptococcus* spp., *Bacteroides fragilis*). Licorice addition showed the largest zones of inhibition compared with the other two substances. The highest inhibition zones in groups with Licorice were against *E. coli* followed by *S. ß haemolyticus.* The paper only stated that AH Plus™ was mixed with each of these compounds, but it did not provide the proportions and amount of each plant extract mixed with the sealer. In order to establish the antibacterial effectiveness of these herbal extracts, further research is needed. Furthermore, the possible impact on the physicochemical properties of sealers should be evaluated.

#### 3.1.3. Iodoform

This organoiodine compound with a chemical formula CHI_3_ has limited use in endodontics due to its possible tooth discolouration potential [64] and toxic effects [65,66,67]. Although, it is known to have great radiopacifying characteristics [68] and is used to fill the root canals of primary teeth [69]. The effect of addition of iodoform into Sealer 26™ is discussed in Section 4.

#### 3.1.4. Calcium Hydroxide

In 1920, Hermann introduced calcium hydroxide for root canal fillings. This inorganic compound with a chemical formula Ca(OH)_2_ is a well-known antibacterial drug used in endodontics. High pH around 12.5 alters enzyme activity and cellular metabolism; hydroxyl ions in an aqueous solution act as highly oxidative free radicals, causing cell membrane damage, protein denaturation, and inhibition of bacterial DNA replication [70]. Sealer 26™ is a commercial epoxy resin-based calcium hydroxide containing sealer. There are publications evaluating various antibacterial as well as physicochemical properties of this material. In this review we mainly focus on in-vitro modifications of commercial epoxy resin sealers. Papers on the influence of AH Plus™ modified by the addition of 5% and 10% w/w of calcium hydroxide on physicochemical properties [45,46] are discussed in Section 4.

### 3.2. Antibiotics

Studies have evaluated the effect of addition of antibiotics (amoxicillin, doxycycline or a triple antibiotic paste/mixture (TAP/TAM) of minocycline, metronidazole and ciprofloxacin in equal proportions [24] or doxycycline, metronidazole and ciprofloxacin in the proportion 1:1:1.25 [25], respectively) in AH Plus™ and/or AH 26™. One study showed that the addition of 1% amoxicillin did not have any significant effect on a 2 week biofilm of *E. faecalis* up to 48 h [23]. In this study, direct contact was established between the biofilm and the root canal sealers, and a model used was close to the clinical situation, wherein the root canal sealer possibly entombs the remnant bacteria within the dentinal tubules and lateral/accessory canals. AH Plus™ and AH Plus™ with 1% amoxicillin presented significantly higher antibiofilm activity compared with the control group, but without any difference between each other.

Antibiofilm test results of the sealers that were used in the experiment two days after mixing and stored for 15 h in contact with the biofilm were not different between each other and the control [23]. However, when the sealers were in contact with the biofilm for 15 h, there was no difference between any of the treatment groups, implying that the antibiofilm activity was only of a short duration. This could have been because of the composition of the sealer, which may offer intrinsic antibacterial properties, and once the sealer set, there was no antimicrobial activity. For the groups with amoxicillin, the short-lived antimicrobial activity may be because of two reasons: (i) the concentration of the drug was minimal and (ii) the drug demonstrated a burst release from the sealer during the setting phase, and after setting, it could not be released. This specific study did not test concentrations of amoxicillin >1%, as it was detrimental to the rheological properties of the sealer [23].

In another study where amoxicillin (10% w/w) was blended with AH Plus™ [47], it was shown that growth of planktonic *E. faecalis* was inhibited up to 1 week, with no significant difference between the time periods (1 day, 3 days or 7 days after mixing of the sealer). However, this study used the DCT and antimicrobial effect on planktonic cells as opposed to a biofilm. Given this, the results should be viewed with caution. Considering this, even after 7 days AH Plus™ with 10% amoxicillin continued to inhibit *E. faecalis* growth. It would be interesting to examine the duration for which this combination will demonstrate antimicrobial activity. However, the addition of such a high concentration of amoxicillin may alter the flow properties (viscosity) of the sealer [23], thereby reducing its penetration into the accessory anatomy of root canals where such residual antimicrobial activity is pertinent.

The addition of different concentrations of amoxicillin or doxycycline (1%, 5%, 10%, 25%, 50% by mass) in AH 26™ sealer demonstrated significantly larger zones of inhibition compared with pure AH 26™, against *E. faecalis* (ATCC 29212) in an agar diffusion test [28]. All concentrations of amoxicillin showed significantly larger inhibitory zones compared with doxycycline. As mentioned above, the agar diffusion test is not a robust method of antimicrobial or antibiofilm testing and only offers preliminary data. The authors of this study also demonstrated bacterial killing within the dentinal tubules in a root canal infection model. This was a valid methodology wherein the authors formed 4 weeks old biofilms of *E. faecalis* in the root canals. Using routine microbiological culture-based techniques (colony forming units), this study showed some very interesting findings. In the 48 h treatment group, AH 26™ demonstrated more bacterial killing in the dentinal tubules, compared with AH 26™ + doxycycline and AH 26™ + amoxicillin. However, after 7 days of treatment, the doxycycline and amoxicillin groups showed no bacteria while the AH 26™ group showed bacterial presence, which was not significantly different from the results of this group in the 48 h biofilm. This was explained by the fact that AH 26™ releases formaldehyde from its hexamethylenetetramine component, during its setting phase and in the initial set mass, which worked in synergism with the antibiotics to kill bacteria. Once the sealer set completely, there was either little or no release of formaldehyde. Formaldehyde is a bactericidal agent, affecting most of Gram-positive, Gram-negative bacteria and fungi [71]. It is present in virtually all cells in the human body as a metabolic by-product. According to the manufacturer, AH Plus™ does not contain formaldehyde as an ingredient. However, during its setting reaction, minimal levels of 3.9 ppm formaldehyde were detected in one study [72]. Such a low level of formaldehyde is similar to its endogenous concentration in human plasma [73,74,75,76,77] and several dozen times lower than the bactericidal level against *E. faecalis* [78].

Notably, one study [25] demonstrated that the addition of 10% (by mass) of triple antibiotic mixture (TAM, consisting of doxycycline, metronidazole and ciprofloxacin in the proportion 1:1:1.25) into AH Plus™ showed an increased percentage reduction in the colony forming units of a standard reference strain of *E. faecalis*. However, it is unknown if this additive influences the physicochemical properties of epoxy resin-based sealers. Ordinola-Zapata et al. showed on the intraorally infected dentine model that the triantibiotic paste (minocycline, metronidazole and ciprofloxacin) was most effective at eradicating the biofilms of bacteria compared with calcium hydroxide and 2% chlorhexidine gel [79]. However, minocycline (a broad-spectrum tetracycline antibiotic) has the potential to cause tooth discolouration [80,81]. As an additive to root canal sealers inside a triple antibiotic paste, this undesirable effect of members of the tetracycline antibiotics group should be taken into consideration.

There is also evidence to demonstrate that AH 26™ mixed with TAP (minocycline, metronidazole and ciprofloxacin in equal proportions) at 10% of the sealer’s total weight kills significantly more bacteria than AH Plus™ mixed with the same drug [24]. One interesting finding is that AH 26™ with TAP showed smaller inhibition zones compared with amoxicillin-containing AH Plus™. However, these results were based on the agar diffusion test. Among the experimental groups mixed with antibiotics, only AH Plus™ with amoxicillin showed no inhibitory zones after 7 days. The same study also showed that incorporation of nanosilver particles (the particle size was not mentioned in this publication) in either AH Plus™ or AH 26™ (10% w/w) did not improve the antimicrobial activities of both sealers. One important aspect that was not explored in this work was the confirmation of the release of nanosilver from the set sealer cements. Also, the addition of antibiotics may affect the properties of sealers and has potential complications such as an allergic host response and antibiotic resistance [82].

### 3.3. Antifungal Drugs

Antifungal drugs, such as ketoconazole and fluconazole, were incorporated separately (0.5% by weight) into AH Plus™ and Sealer 26™. Antifungal activity was evaluated by the radial diffusion technique on agar against 30 clinical and standard reference strains of *Candida albicans*. Both ketoconazole and fluconazole significantly improved the antifungal activity of AH Plus™ and Sealer 26™ after 24 h and 48 h with no significant differences between the two drugs. However, the authors also explained the limitations of the technique used, wherein dental materials with better diffusion ability may show bigger inhibition halos. The authors attempted to minimize the risk of diffusion of the chemicals through the agar before incubation by a pre-incubation period by leaving the plates for 2 h at room temperature [48]. Nevertheless, these manipulations may not prevent errors in evaluating the results.

### 3.4. Nanoparticulate Drugs and Nanoparticle-Based Delivery Systems

Attempts have been made to modify sealers with nanoparticulate drugs with an aim of better penetration into the dentinal tubules as well as into microbial cells for better killing. Furthermore, achieving temporal-controlled release is possible with such nano-encapsulated delivery systems. The nanoparticle-based systems that have been tested as additives into epoxy resin-based sealers for this purpose thus far are chitosan, silver vanadate, quaternary ammonium epoxy silicate (QAES), quaternary ammonium polyethylenimine (QPEI) nanoparticles.

#### 3.4.1. Chitosan

One study reported that chitosan nanoparticles (1 g/150 mg) improved the antibacterial properties of ThermaSeal Plus™ (TS Plus™) epoxy resin-based sealer significantly, compared with the unmodified sealer [21]. Direct contact and membrane-restricted tests were used to evaluate antibacterial properties against *E. faecalis*. The filter membrane reduced the antimicrobial ability of the sealer significantly.

The results of this paper may be considered robust due to the methodology employed, and because several tests were used to ascertain the results. In addition to the DCT and membrane-restricted tests, a root canal model was also used. The root canals were first treated with distilled water, carboxymethyl chitosan (CMCS), or CMCS with Rose Bengal (CMCS/RB; C_20_H_2_Cl_4_I_4_Na_2_O_5_). The latter group was irradiated with light (photodynamic therapy). After that, all specimens were filled with calcium hydroxide solution for 15 min at 37 °C to promote biomineralization. The specimens were then filled with sealers with or without CNps (chitosan nanoparticles) and thermoplasticized gutta-percha. Samples were aged for 1 and 4 weeks and infected with *E. faecalis* for 7 days. Confocal laser scanning microscopy revealed a high total biovolume (considerable bacterial colonization) at the sealer–dentine interface after 1 week of samples ageing with a significantly lower volume of live cells, which means that the TS Plus™ sealer effectively killed bacteria irrespective of the presence of CNps. After 4 weeks of ageing, samples filled with the original TS Plus™ sealer showed high levels of total and viable biovolume of bacterial colonization, but in chitosan groups, these parameters were significantly lower. This indicates that chitosan nanoparticles were able to prevent re-colonization of *E. faecalis* [21].

#### 3.4.2. Silver Vanadate

Nanowires of silver vanadate (AgVO_3_) with an average diameter of 150 nm and micrometre-order length were coated with silver nanoparticles (semispherical in shape and 25 nm in size). Minimum inhibitory concentration (MIC) results were reported to be 500 μg/mL for *E. faecalis* and 31.25 μg/mL for *P. aeruginosa* and *E. coli*. The addition of silver vanadate into AH Plus™ did not appear to demonstrate any significant influence on the inhibition zones against the Gram-positive coccus *E. faecalis* (similar zones of inhibition 7.19 ± 0.71 mm to 8.69 ± 1.00 mm). The results were similar regardless the time of incubation (48 h or 7 days) and the concentration of 0% (without addition), 2.5%, 5%, or 10% of nanowires of silver vanadate in the sealer. Different results were observed for Sealer 26™, while in the 2.5% group the antimicrobial effect was not improved after both 48 h and 7 days, 5% concentration increased the effect, and 10% concentration improved it even more (both significantly, but with no difference between 48 h and 7 days groups). Interestingly, AH Plus™, Sealer 26™, and their modifications with silver vanadate (except 10%) showed no inhibitory effects against the common nosocomial Gram-negative pathogenic bacteria, *P. aeruginosa* and *E. coli.* Only 10% of silver vanadate in AH Plus™ provided zones of inhibition against *P. aeruginosa* (5.83 ± 0.52 mm to 6.30 ± 0.24 mm), while Sealer 26™ showed no inhibition zones in all groups with this bacterium. In the case of E. coli, both sealers had similar results (5.77 ± 0.49 mm to 5.80 ± 0.43 mm for AH Plus™ and 6.00 ± 0 mm for Sealer 26™), which were not statistically significant between the groups of 48 h and 7 days, while all other AH Plus™ and Sealer 26™ (with AgVO_3_ 0%, 2.5%, and 5%) groups did not show any inhibition zones [50].

Another study by Vilela Teixeira et al. [52] where DCT was used, reported no difference in CFU reduction for freshly mixed sealers between all modified groups and pure AH Plus™. The same conclusions were drawn for Sealer 26™. As it was mentioned, freshly mixed epoxy resin sealers possess inherent antibacterial activity. Total inhibition of *E. faecalis* was observed. When the set sealers were tested, the results for modified AH Plus™ were not significantly different from the pure AH Plus™ control. However, in groups of modified Sealer 26™ at concentrations of 5% and 10%, there was a significantly greater inhibition of *E. faecalis.* Epifluorescence microscopy images in set sealers revealed more viable bacteria in groups without nanomaterial.

It can be concluded that only higher concentrations of this material (though not in all cases) were effective against the tested microorganisms. When the results are obtained by the agar diffusion test, more clinically relevant methods need to be implemented in future research to establish antimicrobial effectiveness of the material. Other concerns are cytotoxicity of this silver-based material as well as the potential tooth colour change. For clinical application, these properties need to be estimated and weighed against the possible benefits.

#### 3.4.3. Quaternary Ammonium Epoxy Silicate (QAES)

Gong et al. [49] synthesized quaternary ammonium epoxy silicate (QAES) particles, which proved to be spherical in shape and around 120 nm in diameter with a rough surface. Epoxy groups of these particles give the possibility to copolymerize QAES with epoxy resin materials’ polymer network. In this study, epoxy resin-based sealer, AH Plus™, was incorporated with QAES particles in concentrations of 0%, 2%, 4%, and 8%, then water-aged for 4 weeks. After that, antibacterial activity was evaluated in vitro by the direct contact test (DCT) against *E. faecalis* ATCC 29212 (American Type Culture Collection, Manassas, VA, USA). AH plus™ samples without incorporation of QAES did not inhibit bacterial growth compared with the control group. AH Plus™ groups with the addition of QAES in different concentrations led to the inhibition of bacteria, which was statistically significant compared with AH Plus™ alone and control groups. Moreover, *E. faecalis* biofilm (grown for 7 days) viability was tested by 3D image analysis of live/dead-stained biofilms using confocal laser scanning microscopy. The significant decrease of live bacteria in biofilms was shown and it depended on QAES concentration with fewer numbers of live bacteria in higher concentrations of QAES (4% and 8% by mass) in AH Plus™ sealer.

Thus, immobilization of QAES into an epoxy resin-based sealer by copolymerization of its epoxy groups promotes antimicrobial activity even after 4 weeks due to the antibacterial alkylammonium chain, which is prevented from leaching from the cured epoxy resin-based sealer (AH Plus™) [49].

#### 3.4.4. Quaternary Ammonium Polyethylenimine (QPEI) Nanoparticles

Kesler Shvero et al. [54] developed a new testing method, which provided attraction and killed bacteria under “antigravitational” conditions, i.e., *E. faecalis* to the epoxy resin-based surface (AH Plus™) incorporated with 2% (by mass) of QPEI nanoparticles. The membrane potential of bacteria was measured after 30 min, and the values were significantly reduced (compared with the unmodified AH Plus™) to almost zero, which indicated damage to the bacterial cell membranes. Incorporation of 2% QPEI did not alter the surface topography and material’s hardness. An extremely interesting finding was that the inner layers of the sealer contained the highest concentrations of QPEI, despite which inhibition of bacteria occurred on both surfaces. Fourier Transform Infrared Spectroscopic (FTIR) analysis did not detect any release of QPEI nanoparticles from AH Plus™. Despite the lack of release, confocal microscopic analysis using Live/Dead staining demonstrated red staining of the biofilm mass in all layers, including distant ones, suggesting the effective killing of *E. faecalis*. Numerous green-stained bacteria were found on the surface of the unmodified sealer [54]. Indeed, the green-stained microbes using the Live/Dead stain could indicate live or dead bacteria, but a clear distinction is not possible. However, the red-stained cells indicated dead bacteria [83,84].

There is evidence to show that incorporation of 1.5% QPEI improved the bacterial reduction of the epoxy-amine resin endodontic sealer RCS™ by at least 6 logs, compared with the unmodified sealer [55]. Scanning electron microscopy showed changes in bacterial cell morphology and dispersion without signs of cell division in both the modified and unmodified sealer groups. Addition of QPEI caused lysis of bacteria and the formation of syncytium-like cells. Early biofilm formation with unchanged membranes and dividing cells was seen in the unmodified sealer group. In addition, the antibacterial effect of the medium in which the sealers were placed was established. There was no difference with the control elutes’ values. It could mean that the sealers did not release the antibacterial components after setting. Consequently, it also indicates that the antibacterial properties are attributed to direct contact of bacteria with the sealer. That said, ADT is not a reliable test because: a) it is unable to differentiate between bacteriostatic and bactericidal effects of materials, b) of the fact that antibacterial effect is attributed to the diffusion ability of tested materials in agar. No zones of inhibition were observed for both sealers, confirming a false-negative response [55]. Addition of QPEI nanoparticles to *E. faecalis* suspension decreased the bacterial viable counts after 12 min with no viable bacteria remaining after 1 h. SEM images showed numerous QPEI nanoparticles close to *E. faecalis* cell walls. At pH 5.0, there was a minimal antibacterial activity (viable counts were similar to the values of the control group), while at pH > 5 and pH < 5 antibacterial effect was higher (viable counts were lower than the control group) [55].

Using the direct contact test (DCT), the effect of the addition of QPEI nanoparticles to AH Plus™ was tested against three strains of *E. faecalis* (ATCC 29212 and two wild strains isolated from root canals—RW35 and RN44) [56]. AH Plus™ and AH Plus™ with 1% or 2% w/w concentration of QPEI were exposed to bacterial suspensions for 10 min, 30 min, and 60 min, immediately or 7 days after mixing. In the freshly prepared sealers, antibacterial activities remained largely uninfluenced by the addition of QPEI. QPEI incorporation in either 1% or 2% did not improve significantly the antibacterial effect of AH Plus™ after 7 days of setting. The original AH Plus™ sealer lost much of its antimicrobial properties after 7 days of setting. Total bacterial eradication was possible after a 60 min contact time with the RW35 strain only. This effect was achieved for all the three groups of sealers: AH Plus™ alone or with QPEI 1% and 2% [56].

In contrast to the above-mentioned study, the results of Kesler Shvero et al. [53] demonstrated total bacterial growth inhibition in AH Plus™ samples with 2% w/w QPEI nanoparticles after 4 weeks of ageing. DCT revealed total growth inhibition of up to 6 logs in viable counts in the AH Plus™ + QPEI 2% group. The addition of 0.5% and 1% of QPEI nanoparticles led to a 5 log decrease in count. Significant differences were noted between the commercial sealer and modified sealers. The main reasons for such differences in the results may be attributed to the study methods. Optical density values were used to establish bacterial viability by Kesler Shvero et al., but QPEI nanoparticles themselves can add to optical density values. Further, the authors suppose that different batches of AH Plus™ sealers could also produce different results in this regard [56]. As in the previous study by the same group, ADT did not reveal any antimicrobial effect [55]. This study also showed similar results, as follows. After contact with bacteria with a surface of the unmodified sealer, SEM revealed normally dividing cells and an indication of early biofilm formation. After 60 min of direct contact with a surface of the modified sealer (2% group), bacterial cell wall damage, lysis, no signs of cell division together with the aggregation of bacteria and syncytium-like cell wall fusion were seen by SEM.

Based on the results obtained from previous studies, another study [57] evaluated the effect of additional variants of QPEI nanoparticles that were incorporated in dental materials including AH Plus™ in concentrations 0.5%, 1%, 2% for antibacterial tests (DCT) against *E. faecalis* [57]. QPEI nanoparticles were used as a platform for preparing several QPEI variants as follows: (1) low carbonate QPEI with a minimal amount of (i) NaHCO_3_ (QPEI-LC); (ii) hydrochloric acid (QPEI-Cl); (iii) phosphoric acid H_3_PO_4_ (QPEI-Ph); (2) QPEI nanoparticles with surfactants sodium lauroylsarcosine (QPEI-NLS); and (3) QPEI with glycerol monostearate (QPEI-GMS). The QPEI nanoparticles were incorporated into three epoxy resin-based sealers (AH Plus™, AH 26™, BJM RCS™). Only QPEI-GMS could not be incorporated into the sealers due to its physicochemical properties (fibre-like consistency which did not allow it to incorporate into the materials). The DCT test revealed a total inhibition of *E. faecalis* in groups of 2%, immaterial of the sub-type, while 0.5% and 0% groups did not show any antibacterial activity. The strongest antimicrobial effect was observed in groups with QPEI-NLS nanoparticles. The minimum inhibitory concentration of QPEI-NLS for the total inhibition of *E. faecalis* was 2–4 times lower compared with the original QPEI nanoparticles. SEM analysis showed that in groups of AH Plus™ with QPEI-NLS, only a few minutes of contact with *E. faecalis* resulted in morphologic changes in the bacterial cell membrane, with only a few groups of bacteria found on the surface.

The antibacterial effect of AH Plus™ with 1% QPEI in the dentinal tubules of extracted human teeth with CLSM revealed that the percentage of live cells in the dentinal tubules of the original AH Plus™ group was 77% ± 15% with only 45% ± 13% in the AH Plus™ with the QPEI 1% group [58].

One study investigated the strain-dependent susceptibility by treating biofilms of two strains of *E. faecalis* (ATCC 29212 and RW35, isolated from a root canal with post-treatment apical periodontitis) with AH Plus™ containing 2% w/w QPEI [59]. Biofilms were grown on filter paper discs or cellulose nitrate membrane filters. The direct contact test and membrane-restricted tests were performed together with the crystal violet assay. Both unmodified and modified AH Plus™ showed low optical density values indicating more bacterial inhibition, but only AH Plus™ with 2% QPEI showed significantly lower values than the control groups against the standard reference ATCC strain. No material was effective in killing the bacteria of RW35 strain in membrane-restricted test [59].

## 4. Effect of Antimicrobial Additives on Physicochemical Properties of Epoxy Resin Sealers

BAC and CPC release from AH Plus™ sealer incorporated with 2% by mass of either BAC or CPC was measured by means of HPLC analysis [38]. The amount of the CPC released after week 1 and week 4 was higher than the release of BAC. Also, the release at week 4 was higher than that at week 1 for both BAC and CPC. The differences in both cases were statistically significant. The authors suggested that the higher amount of released CPC is possibly due to “smaller and less rigid molecules” of this compound, being able to diffuse easier from hardened materials.

Although compressive strength is not a commonly tested property for endodontic sealers, the authors explained that determination of the compressive strength may be extrapolated to the extent of alteration of properties by the addition of BAC or CPC. With the addition of CPC (2% w/w) into AH Plus™, the compressive strength increased significantly, while in case of BAC addition (also 2% w/w), the values decreased. This result demonstrates that these additives obviously interfere with the setting chemistry. Quaternary ammonium compounds contain amine groups, which can be beneficial in setting the chemistry of epoxy resin-based materials, such as AH Plus™. In the case of CPC, the setting process was improved, though there was an opposite effect with the addition of BAC.

Using Fourier transform infrared spectroscopy (FTIR) analysis of AH Plus™ sealer modified with 1%, 2%, and 3% of BAC, Arias-Moliz et al. showed high peaks of amines and chlorides [40]. As was explained above, amines initiate the setting reaction. The authors explained the retardation of the setting by the prolonged presence of amines in AH Plus™ with BAC. Also, the setting time of AH Plus™ with BAC steadily increased when a higher concentration of BAC was added into the sealer. Furthermore, the addition of BAC appeared to have a plasticizing effect on the sealer. Notably, the compressive strength of AH Plus™ varied with the additives. Despite both being amines, BAC decreased the strength while CPC increased the strength of AH Plus™. This result was speculated to be due to molecular differences between BAC and CPC.

Flow decreased gradually from pure AH Plus™ to AH Plus™ with 1%, 2%, 3% BAC, though the values were within the requirements of ISO 6876:2012. Solubility values were not different among all groups (in the range from 0.088 ± 0.04 to 0.16 ± 0.11% of weight reduction) [40]. Arias-Moliz et al. also tested properties, which are not usually reported in the literature for root canal sealers: micro-hardness and the contact angle of the pure AH Plus™ and AH Plus™ modified with BAC 1% w/w, 2% w/w or 3% w/w [40]. The contact angle influences the wettability of the material. The contact angle of pure AH Plus™ and AH Plus™ with 1% BAC was close to 86 degrees, while the contact angle of AH Plus™ +2% and +3% BAC was around 59.9 to 61.76, which is significantly lower and provides better wettability of the surface, which means that the surface is more hydrophilic. Such a surface was also suggested to be anti-biofouling owing to the electrostatic repulsion that induces steric changes on the surface. Consequently, the “anti-repellent” effect against bacteria could be higher in these modified sealers. Additionally, a low contact angle will promote better penetration of the sealer into spaces of the root canal system which can improve antibacterial efficacy in dentine. As for micro-hardness measurements, this property provides information on monomer conversion. Micro-hardness values of AH Plus™ with 3% BAC decreased by 75%, and by 50% for 2% BAC, compared with the unmodified AH Plus™. The addition of BAC promotes delayed polymerization and makes the consistency of the sealer softer. Nevertheless, neither contact angle nor micro-hardness measurements are requirements in ANSI/ADA or ISO standards for the endodontic sealer [40].

Considering the physical properties of AH Plus™ with chlorhexidine (CHX) and cetrimide (CTR), Ruiz-Linares et al. [32] investigated the effect of CHX (1% or 2%) and CTR (0.1%, 0.2%, 0.3%, 0.5%) added into AH Plus™ sealer separately or in combination, on physicochemical properties (setting time, flow, solubility, and radiopacity). They found that all combinations produced results within the standards set by ANSI/ADA Specification No. 57. CTR increased setting time, while CHX alone or combined with CTR lowered it. The addition of CTR reduced the flow, but CHX increased it. When CHX and CTR were combined, the flow values were generally not statistically different from those of AH Plus™ alone. Solubility values were in accordance with those stipulated by the ANSI/ADA, indicating that addition of these antimicrobial agents was not detrimental to the resin sealer. In this study, AH Plus™ with 1% CHX and AH Plus™ with 2% CHX had the minimum values of 6.43 ± 0.04 and 6.45 ± 0.07 mm of Al, respectively, while AH Plus™ alone had 6.8 ± 0.03 mm of Al. The ANSI/ADA stipulates that radiopacity values for endodontic sealers should be more than 3 mm of Al. The maximum values were in groups with CTR (with no statistically significant differences among them) with 7.14 ± 0.05 mm of Al being the highest value for AH Plus™ + 0.5% CTR. Combinations of these two chemicals with AH Plus™ showed intermediate values for radiopacity [32].

Only 10% w/w addition of dimethylaminododecyl methacrylate (DMAHDM) into AH Plus™ caused the physicochemical properties to not comply with ANSI/ADA Specification No. 57. Setting time, flow, solubility, and dimensional change of all other concentrations of DMAHDM —2.5% and 5%—did not alter the properties as well as 0.05% w/w, 0.10% w/w and 0.15% w/w addition of nanosilver (NAg). The same results were observed in the combination of both DMAHDM at 2.5% and Nag 0.15% w/w [41].

Physicochemical properties after the addition of hinokitiol into AH Plus™ sealer remained within the ISO requirements for endodontic sealers. The setting time of AH Plus™ with hinokitiol 0.2% increased to some extent. The working time did not show a substantial change. The flowability values were 18.05 ± 3.48 mm for AH Plus™, with 0.2% hinokitiol and 21.62 ± 1.85 mm for the pure AH Plus™ (ISO 6876:2001 requires it to be >20 mm, though a new ISO 6876:2012 set it to be >17 mm). It can be seen that the flow decreased with the addition of hinokitiol, which could be related to the higher viscosity of the modified sealer, though the values are still within the limits of the standard requirements. Film thickness increased with the addition of 0.2% hinokitiol to AH Plus™, but not significantly with both values conforming the requirements (<50 μm). Solubility values of AH Plus™ (0.13 ± 0.06%) increased to 0.24 ± 0.03% with the addition of 0.2% hinokitiol, which was a significant change, yet both values were still in accordance with ISO 6876:2001 requirements (<3%) [42].

Duarte et al. tested AH Plus™ with calcium hydroxide (CH) added to the sealer at 5% and 10% w/w. The level of pH was significantly higher, with a higher percentage of CH (0% < 5% < 10%) at 24 h, 48 h, and 7 days. At 14 days and 30 days, no difference was observed between the 5% and 10% groups, which showed higher pH levels than in pure AH Plus™ (significant results). The Ca^2+^ release values were calculated in mg dL^−1^ with the help of an atomic absorption spectrophotometer at the same time periods. The results were constant and statistically significant within each time point between 10%, 5%, and 0% (pure AH Plus™) groups with the highest amounts of calcium ions released in 10% groups and the lowest in pure AH Plus™ [45]. Calcium ion release may improve mineralization and enhance biological properties. High pH, which represents high alkalinity, promotes increased antibacterial potential and also contributes to the deposition of mineralized tissue [45]. However, it has been reported that calcium hydroxide is dissolved out of the sealers in the long-term, and this may contribute to the root canal filling leakage followed by reinfection and endodontic treatment failure [85,86].

Another study by Duarte et al. with the same materials reported no deterioration in radiopacity and setting time values [46]. AH Plus™ modified with 10% CH showed the lowest flow values, which were different significantly from the other two groups, though all values were higher than 20 mm. Film thickness values of both 5% and 10% modified sealers (close to 70 μm) did not comply with ANSI/ADA Specification No. 57, which requires them to be >50 μm. Dimensional changes were recorded to be positive (from +0.57 ± 0.10% to +1.14 ± 0.26%) which represents an expansion of the materials. These values were not significantly different among each other [46].

Sealer 26™ 1.1 g of powder was added to 1.0 g of resin and mixed during 30 s. Three proportions of iodoform were mixed with the sealer: 1.1 g, 0.55 g, 0.275 g (each group contained powder: resin proportion as 1.1:1.0). Flow values were not different statistically with >20 mm in all groups including pure Sealer 26™. The same was reported for calcium release. The pH values reduced with time and were not statistically significant among groups. Only after 24 h, groups containing 1.1 g and 0.55 g resulted in pH values lower than those in 0.275 g and pure Sealer 26™ groups. Setting time values deteriorated significantly with the addition of iodoform. Also, solubility values were not satisfactory (being from 9.38% to 10.98% of the mass loss of the modified sealers), while pure Sealer 26™ resulted in only 2% mass loss, which is a significant difference [44]. Solubility and setting time deteriorated significantly, which makes it doubtful to use such a modified product in a clinical situation.

Addition of antifungal drugs, ketoconazole or fluconazole, into AH Plus™ and Sealer 26™ by 0.5% w/w did not affect setting time significantly. AH Plus™ + fluconazole had higher flow values (39.00 ± 3.00 mm) than AH Plus™ + ketoconazole (29.00 ± 2.5 mm), though none of these groups had a significant difference compared with pure AH Plus™ values (34.00 ± 3.0 mm). The addition of the drugs into Sealer 26™ had no impact on the significant change of flow values among the tested groups (34.00 ± 3.5 mm to 35.00 ± 2.5 mm). All the physical properties complied with ANSI/ADA Specification No. 57 [48].

Andolfatto et al. [23] showed that addition of 10% w/w amoxicillin into the AH Plus™ sealer was nontoxic on L929 fibroblasts, but physical properties, such as setting time and flow, decreased significantly. Even minimal addition of this antibiotic (~0.25% w/w) showed significant changes of physical properties. AH Plus™ + 0.25%, 0.5%, and 1% amoxicillin showed flow values at least 17 mm (with no statistically significant difference among the groups) which is the minimum requirement by ISO 6876:2012. Pure AH Plus™ showed the highest flow values (22.00 ± 0.73 mm), statistically different from all other groups. Higher proportions of 2.5%, 5.5%, 7.5%, and 10% led to values from 16 to 10 mm which are not recommended for endodontic sealers. The setting time of pure AH Plus™ (730 ± 8.66 min) was significantly higher than the values in the groups where 0.25% (474.2 ± 14.29 min), 0.5% (455.0 ± 5.47 min) or 1% (439.2 ± 8.61 min) amoxicillin was added (w/w) [23]. Several studies have reported the cytotoxic effect of epoxy resin sealers. It should be stated that, whereas unset samples of AH Plus™ showed marked cytotoxicity, this cytotoxicity was either no longer present or significantly reduced in set AH Plus™ [87,88,89,90].

BJM Root Canal Sealer™ is an epoxy resin sealer incorporated with Biosafe^®^ HM4100™. Solomonov and Itzhak [36] compared the physicochemical properties (flow, working time, solubility, and dimensional change) of BJM Root Canal Sealer™ with AH Plus™ and MM-Seal™. Biosafe^®^ HM4100™ is a non-leaching biocide. The flow parameters of all the sealers were within the requirements of the ISO 6876/2012 standards. AH Plus™ exhibited a significantly lower flow (20.18 ± 0.72 mm) than two other sealers. Working time and solubility also conformed to the ISO 6876/2012 recommendations (to be not >3% by mass fraction). However, the dimensional changes of all three sealers did not comply with ISO 6876/2001 standards (mean linear shrinkage not >1%, expansion not >0.1%). AH Plus™ and BJM Root Canal Sealer™ showed values of −2.12 ± 0.91 and −5 ± 2.34, respectively, indicating shrinkage, but there was no significant difference between the two materials. On the other hand, MM-Seal™ had a significantly higher shrinkage compared with the other two sealers (−12.2% ± 3.03%) [36]. Some antibiofilm effects of the addition of this quaternary ammonium compound were demonstrated in [37], although it needs further investigation in more clinically relevant studies together with cytotoxicity evaluation.

The addition of nanowires of silver vanadate (AgVO_3_) decreased the flow of AH Plus™ with 5% and 10% groups having the lowest flow values (24.6 ± 0.18 mm and 18.2 ± 0.08 mm, respectively), while the pure AH Plus™ had 36.2 ± 0.13 mm. No significant changes were observed in Sealer 26™ (all values were above 40 mm). No group had values less than those recommended by ISO 6876:2012 (>17 mm), as was previously mentioned, though ANSI/ADA Specification No. 57 and ISO 6876:2001 require flow values to be >20 mm [50]. Two studies by Vilela Teixeira et al. [50,51] measured radiopacity values. 2.5% groups in both studies for both sealers showed significantly increased values compared with the non-modified samples (with an exception of Sealer 26 in the study [50], where there was no difference observed). Intriguingly, the 10% groups for both AH Plus™ and Sealer 26™ showed lower radiopacity values than the 2.5% group in the study [51], but not in [50], where the results were not different statistically. Within each sealer, almost all 5% and 10% groups had the same values as the 0% groups (except AH Plus™ in study [50] with 10% > 0%, though 10% = 5%). Pure Sealer 26™ in the study [51] had values up to 7.37, though in [50], the same sealer had values around 3.91 ± 0.19 mm Al. Such a big difference could probably be explained with either a different manipulation (e.g., a different liquid to powder ratio) of the sealer or another batch of this product. However, both studies presented radiopacity values (with the minimum being 3.80–4.20 mm and 3.83 ± 0.41 mm Al) higher than the required 3 mm Al for sealers. In general, these differences may be neglected clinically. The authors explained the differences in radiopacity values by the uneven distribution and agglomeration of the material. Furthermore, the addition of 2.5% and 10% caused the most significant discolouration of incisors at 180 days in AH Plus™ groups, and the biggest tooth colour change for Sealer 26™ was with 10% at different assessment times [51], which supports a concern on tooth colour change by silver-based additives. In another study by Vilela Teixeira et al. [52] solubility values of modified and pure AH Plus™ did not differ significantly. The differences in pH values were significant in modified sealers in comparison with pure AH Plus™ (time points 6 h, 24 h, 14 days), but no difference at 7 days and 30 days. Comparisons within the same concentrations of silver vanadate revealed pH variations at the initial time point and last time point of 30 days. All groups showed acidic pH relative to the initial pH. Modification of Sealer 26™ led to small pH variation between groups, except for the 10% group, which showed a difference at 6 h, and the 5% group, which showed a difference at 7 days. Within the same concentration, there was the increase of pH with time—a significant difference at 6 h compared with 7 days, 14 days, and 30 days [52].

The effect of the addition of 1%, 1.5%, and 2% w/w QPEI nanoparticles on the flow and solubility to RCS™, epoxy-amine resin sealer, showed that flow results were lower for modified sealers, but they were within the ISO 6876:2001 specifications [55]. This sealer was the precursor to the BJM Root Canal Sealer™ discussed earlier. All solubility values (from 0.023% to 0.030%) were in accordance with the mentioned specifications. Glass transition temperature (Tg) was also evaluated. Addition of up to 1.5% of QPEI nanoparticles did not change the Tg significantly, which suggests that the setting of the sealer would likely remain unaffected. Incorporation of 2% QPEI nanoparticles brought about a reduction of Tg. While this may be attributed to the addition of nanoparticles, direct conclusions cannot be drawn about its impact on the setting. However, incomplete polymerization was observed in all the samples. The modified sealer was non-cytotoxic in a test with L929 cell cultures [55].

It was also demonstrated [56] that addition of 1% w/w and 2% w/w QPEI did not significantly alter the physicochemical properties of AH Plus™, such as setting time (470 ± 10 to 475 ± 15 min), flow (29.0 ± 0.66 to 27.8 ± 0.21 mm), solubility (0.7 ± 0.1% to 0.8 ± 0.1%), apparent porosity (5.6 ± 1.4% to 6.0 ± 1.3%), and dimensional change (−0.43 ± 0.01% to −0.40 ± 0.01%). There was no significant change in the compressive strength either (7.68 ± 0.41 MPa to 7.56 ± 0.04 MPa). Interestingly, incorporation of QPEI significantly improved the hydrophilicity of AH Plus™. Wettability of AH Plus™ [unmodified (80.9° ± 0.4°) and modified with 1% (65.8° ± 1.9°) and 2% (56.2° ± 2.0°) w/w QPEI] was calculated by contact angle measurement. Zeta potential of the unmodified AH Plus™ was measured to be −8.26 ± 1.27 mV, while for AH Plus™ with 1% QPEI it was −8.12 ± 1.25 mV, and in the case of AH Plus™ with 2% QPEI, the zeta potential was −3.85 ± 0.6. The latter value was significantly higher than the former ones. It could be stated that the surface charge increased with the addition of 2% QPEI. One may reflect that the superior penetration of such hydrophilic sealers may help eradicate residual bacteria in the anatomic irregularities of the root canal system and the dentinal tubules. Furthermore, the positively charged QPEI may be effective against certain bacterial species [54].

## 5. Conclusions

This review confirms a widespread interest in modifying root canal sealers with improved antimicrobial activity. It is apparent that studies use different methodologies to evaluate this outcome, making direct, vis-à-vis comparisons impossible. The use of quaternary ammonium-based compounds and antibacterial agent-functionalised nanoparticles appear to be promising in exhibiting anti-biofilm activities, but the safety of nanoparticles to the human body systems and tissues should be confirmed first before any clinical use. Another problem is that sealers modified with leachable agents can have increased solubility and, consequently, predispose root canal fillings to leakage. It may lead to undesirable outcomes of endodontic treatment. As an example, calcium hydroxide or antibiotics as a component of a sealer may be detrimental in this aspect. Further, topical application of antibiotics is a questionable practice. It may lead to antibiotic resistance of the root canal flora. The mechanism of action of quaternary ammonium compounds is different and is believed to be due to the contact killing but developing microbial resistance is also a possibility, especially in concentrations below minimum inhibitory levels.

Does a root canal sealer need to possess any antibacterial activity at all? This question does not have a definitive answer. There is a strong point of view that bacteria should be eliminated during chemo-mechanical preparation with the following obturation with the goal of achieving a bacteria-tight filling. On the other hand, taking into consideration the difficulties in effectively disinfecting some complexities of the root canal, if endodontic sealers could provide both antibacterial and anti-biofouling properties, they could prevent from the possible multiplication of residual microorganisms.

Another question is the cytotoxicity of epoxy resin-based sealers. As was previously discussed, these sealers are cytotoxic only during setting, with a significant reduction of cytotoxicity and becoming non-cytotoxic after setting. The release of formaldehyde together with amine and epoxy resin components may explain the cytotoxic effect of epoxy-resin containing sealers. A positive correlation between the cytotoxity and antibacterial effect is also observed. The set epoxy resin-based sealers mainly lose their antibacterial activity. Due to the possibility of the cytotoxicity of epoxy resin sealers, the future research should be performed with a good design and proper control groups to differentiate whether the additives to a sealer provide any positive antimicrobial effect.

Based on the studies included in the review, antimicrobial additives in many cases improved the antimicrobial effect of epoxy resin-based sealers which could be observed long after the sealers set. Physicochemical properties often remained in accordance with specifications for this type of materials. The discrepancy between some results could be explained by variation in the conditions of the experiments. The addition of antibacterial agents into root canal sealers can be performed only if it does not deteriorate the properties, damage the root filling’s integrity, and does not pose a health risk to the patient.

Future research should take into consideration all these concerns and compare and contrast the time and concentration-dependent effectiveness of antibacterial agents in inhibiting multi-species endodontic biofilms in robust, clinically relevant models.

## Figures and Tables

**Table 1:** Search strategy with the keywords used in the PubMed database.

Search Builder	Search Words	Results
#1	“epoxy resin” OR epoxy-based OR amine-epoxy OR amine epoxy OR epoxy OR epoxide	41,898
#2	Sealer * OR sealing OR sealant * OR filling * OR cement *	150,150
#3	antibacterial OR antimicrobial OR antibiofilm OR anti-biofilm OR biofilm * OR fungus OR fungi OR fungal OR antifungal OR anti-fungal OR bactericidal OR infection OR antiinfective OR anti-infective OR microbial OR bacterial OR nanoparticle * OR antibiotic *	4,663,057
#1 AND #2 AND #3		656

* Asterisk in Pubmed after a search word leads to finding of all terms that begin with a given string of text.

**Table 2:** Search strategy with the keywords used in Scopus database.

Advanced Search Field in Scopus with Key Words	Results
(TITLE-ABS-KEY (“epoxy resin” OR epoxy-based OR amine-epoxy OR “amine epoxy” OR epoxy OR epoxide ) AND TITLE-ABS-KEY (sealer * OR sealing OR sealant * OR filling * OR cement *) AND TITLE-ABS-KEY (antibacterial OR antimicrobial OR antibiofilm OR anti-biofilm OR biofilm * OR fungus OR fungi OR fungal OR antifungal OR anti-fungal OR bactericidal OR infection OR antiinfective OR anti-infective OR microbial OR bacterial OR nanoparticle * OR antibiotic *))	335

* TITLE-ABS-KEY stands for the “Article title, Abstract, Keywords” field in Scopus.

**Table 3:** General characteristics of studies included in this review.

Publication	Epoxy Resin Sealer Tested	Antimicrobial Additive(s)	Methods of Studying the Antimicrobial Activity	Physicochemical Properties Tested	Main Results
Bailon-Sanchez et al. [31]	AH Plus™	Chlorhexidine digluconate (CHX) liquid 1%, 2% and cetrimide (CTR) 0.1%, 0.2%, 0.3%, 0.4%, 0.5%, and a mixture of both	DCT; Biofilm test: Calgary Biofilm Device (MBEC-high throughput [HTP]; Innovotech, Edmonton, AB, Canada).	Not tested	AH Plus™ with 1% or 2% CHX: higher antibacterial effect than AH Plus™ alone. Inhibition of *E. faecalis* biofilm formation: depends on CHX % in AH Plus™. CTR 0.1% + 1% or 2% CHX, CTR 0.2% inhibited biofilms Eradication concentrations: 0.3%, 0.4%, 0.5% CTR + 2% CHX. CHX alone: no eradication of biofilm.
Ruiz-Linares et al. [32]	AH Plus™	Chlorhexidine digluconate (CHX) liquid 1%, 2% and cetrimide (CTR) 0.1%, 0.2%, 0.3%, 0.5%, and mixture of both	Not tested	Setting time, flow, solubility, radiopacity	All concentrations: results are within ANSI/ADA Specification No. 57. Setting time: CTR—increased; CHX alone or CHX+CTR decreased. Flow: CTR—reduced; CHX—increased; CHX + CTR = AH Plus alone.
Solomonov et al. [36]	BJM Root Canal Sealer™, AH Plus™, MM-Seal™	Biosafe^®^ HM4100™ (3-(Trihydroxysilyl) propyldimethyl-octadecyl ammonium chloride) as an additive into BJM Root Canal Sealer™.	Not tested	Flow, working time, solubility, dimensional change	Flow: all values within ISO 6876/2012. Dimensional change: all values inconsistent with the ISO 6876/2001. Solubility: AH Plus™ and BJM Root Canal Sealer™ in accordance with ISO 6876/2012.
Becker et al. [37]	BJM Root Canal Sealer™	Biosafe^®^ HM4100™ (3-(Trihydroxysilyl) propyldimethyl-octadecyl ammonium chloride) at 0%, 0.4%, 0.8%, 1.6%, 3.3% w/v	Crystal violet staining, Optical density (spectrophotometer). Fluorescence microscopy	Not tested	Significant reduction in de-novo biofilm formation of 25% and 72% in 1.6% and 3.3% *w*/*v* groups, respectively. Biofilm viability: significant reduction of 20% and 36% in 1.6% and 3.3% *w*/*v* groups.
Gjorgievska et al. [38]	AH Plus™	Benzalkonium Chloride (BAC), Cetylpyridinium Chloride (CPC) at 2% w/w	Not tested	Release of BAC and CPC after week 1 and week 4. Compressive strength.	BAC and CPC release: at week 4 higher than at week 1. CPC release: higher than BAC release at week 1 and 4. Compressive strength: CPC increased; BAC decreased.
Gjorgievska et al. [39]	AH Plus™	Benzalkonium Chloride (BAC), Cetylpyridinium Chloride (CPC) at 2% w/w	ADT	Not tested	AH Plus™ + 2% BAC or 2% CPC: zones of inhibition larger than in pure AH Plus™ (*Streptococcus mutans* and *Lactobacillus casei*). Decreased with time. Pure AH Plus™: no inhibition zones (*S. mutans*) after 7 and 21 days; 1% and 2% groups: larger inhibition zones. Best effect: against *Actinomyces viscosus*. Effect decreased after 7 and 21 days.
Arias-Moliz et al. [40]	AH Plus™	Benzalkonium chloride (BAC) at 1%, 2%, and 3% w/w	DCT; Antibiofilm test: MBEC-high-throughput (HTP) device; CLSM	Setting time, flow, solubility, microhardness, contact angle measurement	Strength: CPC increased; BAC decreased. Flow: within ISO 6876:2012, but decreased gradually from pure AH Plus™ to AH Plus™ + 1%, 2%, 3% BAC. Solubility: not different in all groups. Contact angle of AH Plus™ + 2% and + 3% BAC: lower than of pure AH Plus™ or with 1% BAC. Better wettability, more hydrophilic surface. Microhardness: AH Plus™ + 3% BAC decreased by 75%; + 2% BAC decreased by 50%, compared with pure AH Plus™.
Seung et al. [41]	AH Plus™	Dimethylaminododecyl methacrylate (DMAHDM) at 2.5%, 5%, 10% w/w; Nanosilver (NAg) 0.05%, 0.10%, 0.15% w/w; Combination of DMAHDM 2.5% w/w and NAg 0.15% w/w	DCT (modified DCT) CFU counts	Setting time, flow, solubility, dimensional change.	All the tested properties of AH Plus™ with 2.5% DMAHDM and 0.15% NAg did not change significantly. Only 10% DMAHDM caused physical properties to go beyond ANSI/ADA Specification No. 57. The mean CFU/mL for AH Plus™ + 2.5% DMAHDM and AH Plus™ with 2.5% DMAHDM and 0.15% NAg were significantly lower compared with the control at each time point (day 1, day 7, day 14). AH Plus™ with 0.15% NAg had a significant bacterial reduction on day 1 compared with pure AH Plus™, but no difference at days 7 and 14.
Shih et al. [42]	AH Plus™	Hinokitiol at 0.2%, 0.5%, 1%, and 2% w/w 0.2% used for physical, biological and antimicrobial tests	ADT; DCT	Setting time, working time, flowability, film thickness, solubility, cytotoxicity.	ADT: pure AH Plus™-clear inhibition zones [methicillin-resistant *Staphylococcus aureus* (MRSA)]. 0.2% hinokitiol: effect increased. DCT: growth of MRSA inhibited 100% by pure AH Plus™ or + 0.2% hinokitiol. Physicochemical properties: AH Plus™ + 0.2% hinokitiol—within ISO.
Saha et al. [43]	AH Plus™	Herbal extracts: *Glycyrrhiza glabra* (Licorice); *Tinospora cordifolia* (Guduchi); *Mimusops elengi* (Bakul)	ADT	Not tested	AH Plus™ with Licorice showed the highest inhibition zones. Licorice: the highest inhibition zones were against *E. coli* followed by *S. ß haemolyticus.*
Kuga et al. [44]	Sealer 26™	Iodoform at 0.275 g: 2.1 g; 0.55 g: 2.1 g; 1.1 g: 2.1 g (to the sealer)	Not tested	Setting time, flow, solubility, pH, calcium release.	Setting time: deteriorated significantly; Flow: not significant differences, all > 20 mm; Solubility (mass loss): pure AH Plus™ −2%; modified groups −9.38% to −10.98%. pH: only at 24 h significant differences—1.1 g and 0.55 g < 0.275 g and pure Sealer 26™ other groups (7 days, 14 days, 21 days, 28 days, 45 days): not significant differences; Calcium release: not significant differences;
Duarte et al. [45]	AH Plus™	Calcium hydroxide (CH) at 5% w/w and 10% w/w	Not tested	pH, calcium release (Ca^2+^)	pH: pure AH Plus™ < AH Plus™ 5% CH < AH Plus™ 10% CH at 24 h, 48 h, 7 days; pH: pure AH Plus™ < AH Plus™ 5% CH = AH Plus™ 10% CH at 14, 30 days; Ca^2+^ release: pure AH Plus™ < AH Plus™ 5% CH < AH Plus™ 10% CH at 24 h, 48 h, 7 days, 14 days, 30 days
Duarte et al. [46]	AH Plus™	Calcium hydroxide (CH) at 5% w/w and 10% w/w	Not tested	Setting time, flow, film thickness, solubility, dimensional changes, radiopacity	Setting time: not significant differences; Flow: pure AH Plus™ = AH Plus™ 5% CH; AH Plus™ 10% CH significantly lower; all > 20 mm. Film thickness: pure AH Plus < AH Plus™ 10% CH = AH Plus™ 5% CH; Solubility: pure AH Plus™ < AH Plus™ 10% CH; pure AH Plus™ = AH Plus™ 10% CH; AH Plus 5% CH = AH Plus™ 10% CH; Dimensional changes: not significant differences (expansion ranges 0.57 ± 0.1% to 1.14 ± 0.26%); Radiopacity: not significant differences, all groups > 3 mm Al.
Andolfatto et al. [23]	AH Plus™	Amoxicillin added at 0.25% w/w, 0.5%, 1%, 2.5%, 5.5%, 7.5%, 10% w/w into the sealer	CFU counts	Flow, setting time, cytocompatibility	1% amoxicillin: no increase in antibiofilm activity. >1% amoxicillin: physicochemical properties deteriorated. Flow: 0.25%, 0.5% and 1% within the ISO. Setting time: decreased at 0.25% and higher. Cytocompatibility: viability of fibroblasts—not diminished; changes in the cytoskeleton—not affected.
Kangarlou et al. [24]	AH Plus™, AH 26™	Amoxicillin; triple antibiotic paste (TAP); nanosilveradded at 10% w/w into sealers.	ADT	Not tested	Amoxicillin and TAP: improved antibacterial properties. The effect decreased with time. Incorporation of nanosilver did not improve the antibacterial effect.
Vanapatla et al. [25]	AH Plus™	Triple antibiotic mixture (TAM) added at 10% w/w together with a gutta-percha point	CFU counts	Not tested	Antibacterial effect increased significantly (increased percentage reduction in the CFU of *E. faecalis*).
Razmi et al. [28]	AH 26™	Amoxicillin; doxycycline added at 1%, 5%, 10%, 25%, 50% w/w into the sealer	ADT; CFU counts (In vitro human root inoculation method)	Not tested	AH 26™ pure and + 1%, 5%, 10%, 25% and 50% of amoxicillin or doxycycline: zones of inhibition; pure AH 26™: smaller zones. The peak of antimicrobial activity: at 1%. AH 26™ + amoxicillin: larger inhibition zones than AH 26™ + doxycycline in all concentrations. The mean log_10_CFU for AH 26™ doxycycline combination: lower. AH 26™ + amoxicillin and AH 26™ + doxycycline: total eradication of *E. faecalis* in the dentinal tubules after 7 days.
Baer and Maki [47]	AH Plus™	Amoxicillin at 10% w/w	DCT Optical density (spectrophotometer)	Not tested	*E. faecalis* (planktonic) growth: inhibited up to 1 week. No significant difference at 1 day, 3 days or 7 days after mixing. Amoxicillin: improved antibacterial effect (*E. faecalis*) in fresh and set sealers.
Weckwerth et al. [48]	AH Plus™, Sealer 26™	Ketoconazole; Fluconazole at 0.5% w/w	ADT	Setting time, flowability	Antifungal effect (*C. albicans)* improved significantly. Setting time: not changed significantly. Flow: not changed significantly compared with pure sealers. All results: within ANSI/ADA specification No. 57.
Del Carpio-Perochena et al. [21]	ThermaSeal Plus™	Chitosan nanoparticles (CNps)	CFU calculation (log CFU/mL); Direct contact and membrane-restricted antibacterial experiments; CLSM, Sealer–dentine interface after pretreatment of dentine with CMCS (carboxymethyl-chitosan) or CMCS+RB (rose bengal)	Not tested	CNps improved antibacterial properties significantly. After 1 week: antimicrobial activity was higher regardless of CNPs presence. After 4 weeks: bacterial colonization was higher. Chitosan group: significant reduction in total and viable biovolume.
Vilela Teixeira et al. [50]	AH Plus™, Sealer 26™	Nanostructured silver vanadate (AgVO_3_) decorated with silver nanoparticles at 0% w/w, 2.5% w/w, 5% w/w, 10% w/w	MIC (for AgVO_3_) by the visual assessment of turbidity; ADT	Flow, radiopacity	MIC: 500 μg/mL for *E. faecalis*; 31.25 μg/mL *for P. aeruginosa* and *E. coli*. *E. faecalis*—AH Plus™: 0% = 2.5% = 5% = 10% inhibition zones, *E. faecalis*—Sealer 26™: 0% = 2.5% < 5% < 10%. *P. aeruginosa*—AH Plus™: 0% = 2.5% = 5% no inhibition; 10% inhibition zones. *P. aeruginosa*—Sealer 26™: 0% = 2.5% = 5% = 10% no inhibition. *E. coli:* AH Plus™ and Sealer 26™ same results— 0% = 2.5% = 5% no inhibition, 10% inhibition zones. Flow: AH Plus™-decreased; Sealer 26™ not changed. Radiopacity: all values > 3 mm Al (within ANSI/ADA No. 57).
Vilela Teixeira et al. [51]	AH Plus™, Sealer 26™	Nanostructured silver vanadate (AgVO_3_) decorated with silver nanoparticles at 0%, 2.5%, 5%, 10% w/w	Not tested	Radiopacity, colour change	Radiopacity: all values > 3 mm Al (within ANSI/ADA No. 57). Most significant discolouration of teeth at 180 days. Biggest change: AH Plus™ 2.5% and 10%; Sealer 26™ 10%.
Vilela Teixeira et al. [52]	AH Plus™, Sealer 26™	Nanostructured silver vanadate (AgVO_3_) decorated with silver nanoparticles at 0%, 2.5%, 5%, 10% w/w	DCT CFU counts; Epifluorescence microscopy	pH, solubility	Fresh sealers: both pure and modified AH Plus™ and Sealer 26™ provided total inhibition of *E. faecalis* in DCT. Set sealers: modified AH Plus™ showed no difference with pure AH Plus™ in all concentrations tested in DCT. Sealer 26™ 5% and 10% groups provided significantly higher inhibition of *E. faecalis* compared with pure Sealer 26™ in DCT. Epifluorescence images in set sealers showed more viable bacteria in nanomaterial-free groups. Solubility values of modified AH Plus™ and Sealer 26™ did not change significantly. pH: AH Plus™—significant difference in pH with time (6 h, 24 h, 14 days) of the modified groups compared with pure AH Plus™ except time points of 7 days and 30 days. Within the same concentration of AgVO_3:_ pH varied at the initial time point and final time point of 30 days. All groups showed acidic pH in relation to the initial pH. pH: Sealer 26™—small pH variation between groups, except 10% group presenting a difference at 6 h, and 5% group presenting a difference at 7 days. Within the same concentration: increase of pH with time—significant difference at 6 h compared with 7 days, 14 days and 30 days.
Gong et al. [49]	AH Plus™	QAES (quaternary ammonium epoxy silicate) particles at 2%, 4%, 8% w/w	DCT; Optical density (spectrophotometer); Viability analysis of biofilm: CLSM	Not tested	Inhibition of *E. faecalis*: significantly higher compared with pure AH Plus™. CLSM: *E. faecalis* biofilm (grown for 7 days) viability—significant decrease of live bacteria in biofilms, it depended on QAES concentration; less numbers of live bacteria in higher concentrations of QAES (4% and 8%).
Kesler Shvero et al. [53]	AH Plus™	QPEI nanoparticles at 0.5%, 1%, 2% w/w	DCT Optical density (spectrophotometer); ADT; SEM	Not tested	After 4 weeks of ageing: total bacterial growth inhibition in 2% group. DCT: total growth inhibition of up to 6 logs in viable counts in AH Plus + QPEI 2%. 0.5% and 1% of QPEI: 5 logs decrease in count. Significant differences between pure sealer and modified sealers. ADT: no effect—IABN (QPEI NPs) not diffusing into the agar. SEM: bacterial cell wall damage and lysis.
Kesler Shvero et al. [54]	AH Plus™	QPEI Nanoparticles at 2% w/w	Optical density (spectrophotometer); CLSM; Flow cytometry	Not tested	Changes in fluorescence from green to red: increasing membrane potential. Bacterial membrane destabilization and cell death. QPEI NPs: incorporated in the sealer’s surface without damaging its mechanical properties.
Beyth et al. [55]	RCS (BJM)™	QPEI Nanoparticles at 1.5% w/w	DCT; Optical density (spectrophotometer), CFU counts ADT; SEM	Solubility, flow, cytotoxicity	Antibacterial effect of immobilized nanoparticles (QPEI Nps): the number of residual viable bacteria decreased 6 logs vs the number in RCS™ without nanoparticles. SEM: syncytium-like cells and bacterial lysis after contact between the bacteria and a surface with QPEI NPs. Contact of bacteria with pure sealer: early biofilm formation with intact membranes and dividing cells. ADT: no inhibition in all groups. Antibacterial effect of QPEI NPs in suspension: a decrease in viable counts; after 1 h: no viable bacteria. SEM: NPs close to *E. faecalis* cell walls. Adequate physical properties are maintained. Non-cytotoxic.
Barros et al. [56]	AH Plus™	QPEI nanoparticles at 1% or 2% w/w	DCT, CFU counts	Setting time, flow, solubility, apparent porosity, dimensional change, wettability, zeta potential, compressive strength	Total eradication of *E. faecalis*: in fresh samples after 60 min of contact with RW35 strain of *E. faecalis* regardless of the presence of QPEI nanoparticles. 1% or 2% QPEI: no increase in the antibacterial effect of AH Plus™ after ageing. 1% and 2% QPEI: no significant change in physicochemical properties of AH Plus™—setting time, flow, solubility, apparent porosity and dimensional change (within ISO 6876:2001). Compressive strength: no change. QPEI: improved hydrophilicity. Wettability (by contact angle measurement) of AH Plus™: higher in 1% and 2%, lower in pure AH Plus™. Zeta potential: surface charge increased in 2% QPEI group.
Zaltsman et al. [57]	AH Plus™	QPEI nanoparticles variants	DCT, Optical density (spectrophotometer) MIC SEM	Not tested	2% QPEI: total inhibition of *E. faecalis*. 1% QPEI: only a partial effect. 0.5% and 0% QPEI: no effect SEM of pure AH Plus™: normal cells, intact cell wall. SEM QPEI-NLS at 2% w/w: morphologic changes in the bacterial cell wall with only a few bacteria on the surface.
Abramovitz et al. [58]	AH Plus™	QPEI nanoparticles at 1% w/w	CLSM	Not tested	Percentage of live cells in the dentinal tubules of pure AH Plus™: significantly higher compared with AH Plus™ + 1% QPEI. QPEI effect: significant reduction in live bacteria (*E. faecalis*).
Barros et al. [59]	AH Plus™	QPEI nanoparticles at 2% w/w	DCT, CFU counts; membrane-restricted test; Crystal-violet microtiter-plate assay CLSM (data not shown in the paper)	Not tested	Both pure and modified AH Plus™: low optical density values—more bacterial inhibition. Only AH Plus™ + 2% QPEI: significantly lower values (*E. faecalis* ATCC 29212 strain). *E. faecalis* RW35 strain in the membrane-restricted test: no material was effective.

Key: DCT = direct contact test; CLSM = confocal laser scanning microscopy; ADT = agar diffusion test; CFU = colony forming units; TAP = triple antibiotic paste (minocycline:metronidazole:ciprofloxacin 1:1:1); TAM = triple antibiotic mixture (doxycycline:metronidazole:ciprofloxacin 1:1:1.25); MIC = minimum inhibitory concentration; SEM = scanning electron microscopy; QPEI = quaternary ammonium polyethylenimine; NPs = nanoparticles.
